# Genome-wide association analyses of symptom severity among clozapine-treated patients with schizophrenia spectrum disorders

**DOI:** 10.1038/s41398-022-01884-3

**Published:** 2022-04-07

**Authors:** C. Okhuijsen-Pfeifer, M. Z. van der Horst, C. A. Bousman, B. Lin, K. R. van Eijk, S. Ripke, Y. Ayhan, M. O. Babaoglu, M. Bak, W. Alink, H. van Beek, E. Beld, A. Bouhuis, M. Edlinger, I. M. Erdogan, A. Ertuğrul, G. Yoca, I. P. Everall, T. Görlitz, GROUP (Genetic Risk and Outcome of Psychosis) investigators, T. van Amelsvoort, A. A. Bartels-Velthuis, R. Bruggeman, W. Cahn, S. Guloksuz, L. de Haan, R. S. Kahn, F. Schirmbeck, C. J. P. Simons, J. van Os, B. Z. Alizadeh, J. J. Luykx, B. P. F. Rutten, R. van Winkel, K. P. Grootens, S. Gutwinski, T. Hallikainen, E. Jeger-Land, M. de Koning, M. Lähteenvuo, S. E. Legge, S. Leucht, C. Morgenroth, A. Müderrisoğlu, A. Narang, C. Pantelis, A. F. Pardiñas, T. Oviedo-Salcedo, J. Schneider-Thoma, S. Schreiter, E. Repo-Tiihonen, H. Tuppurainen, M. Veereschild, S. Veerman, M. de Vos, E. Wagner, D. Cohen, J. P. A. M. Bogers, J. T. R. Walters, A. E. Anil Yağcıoğlu, J. Tiihonen, A. Hasan, J. J. Luykx

**Affiliations:** 1 Department of Psychiatry, University Medical Center Utrecht, Utrecht University, Brain Center, Utrecht, The Netherlands; 2 Department of Translational Neuroscience, University Medical Center Utrecht, Utrecht University, Brain Center, Utrecht, The Netherlands; 3 GGNet Mental Health, Warnsveld, The Netherlands; 4 Department of Medical Genetics, University of Calgary, Calgary, Canada; 5 Department of Psychiatry, University of Calgary, Calgary, Canada; 6 Department of Physiology & Pharmacology, University of Calgary, Calgary, Canada; 7 Department of Psychiatry, University of Melbourne, Melbourne Neuropsychiatry Centre, Melbourne, Australia; 8 Charité – Universitätsmedizin Berlin, corporate member of Freie Universität Berlin, Humboldt-Universität zu Berlin, and Berlin Institute of Health, Department of Psychiatry and Psychotherapy, Berlin, Germany; 9 Department of Psychiatry, Faculty of Medicine, Hacettepe University, Ankara, Turkey; 10 Department of Pharmacology, Faculty of Medicine, Hacettepe University, Ankara, Turkey; 11 Department of Psychiatry and Neuropsychology, Maastricht University, Maastricht, The Netherlands; 12 Mondriaan, Mental Health Institute, Maastricht, The Netherlands; 13 Multicomplexe Zorg, Pro Persona, Wolfheze, The Netherlands; 14 Clinical Recovery Clinic, Mental Health Services Rivierduinen, Leiden, The Netherlands; 15 Mental Health Organization North‐Holland North location Den Helder, Den Helder, The Netherlands; 16 Program for early psychosis & severe mental illness, Pro Persona Mental Healthcare, Wolfheze, The Netherlands; 17 Department of Psychiatry, Psychotherapy and Psychosomatics, Division for Psychiatry I, Medical University Innsbruck, Innsbruck, Austria; 18 Şarkışla State Hospital, Ministry of Health, Sivas, Turkey; 19 Institute of Psychiatry, Psychology and Neuroscience, King’s College London, London, United Kingdom; 20 Department of Psychiatry and Psychotherapy, University Hospital, LMU Munich, Munich, Germany; 21 Department of Psychiatry, Psychotherapy and Psychosomatics, Medical Faculty University Augsburg, Bezirkskrankenhaus Augsburg, Augsburg, Germany; 22 Reinier van Arkel, s-Hertogenbosch, The Netherlands; 23 Unit for Clinical Psychopharmacology and Neuropsychiatry, Radboud University Medical Centre, Nijmegen, The Netherlands; 24 Department of Forensic Psychiatry, University of Kuopio, Niuvanniemi Hospital, Kuopio, Finland; 25 Arkin, Institute for Mental Health, Amsterdam, The Netherlands; 26 Amsterdam UMC, University of Amsterdam, Department of Psychiatry, Amsterdam, The Netherlands; 27 Division of Psychological Medicine and Clinical Neurosciences, MRC Centre for Neuropsychiatric Genetics and Genomics, School of Medicine, Cardiff University, Cardiff, United Kingdom; 28 Department of Psychiatry and Psychotherapy, School of Medicine, Klinikum rechts der Isar, Technical University of Munich, Munich, Germany; 29 Department of Pharmacology, Faculty of Medicine, Kırıkkale University, Kırıkkale, Turkey; 30 Berlin Institute of Health (BIH), BIH Biomedical Innovation Academy, Berlin, Germany; 31 Mental Health Organization North‐Holland North location Alkmaar, Alkmaar, The Netherlands; 32 Mental Health Organization North‐Holland North location Heerhugowaard, Heerhugowaard, The Netherlands; 33 High Care Clinics, Mental Health Services Rivierduinen, Leiden, The Netherlands; 34 Department of Clinical Neuroscience, Karolinska Institutet, Solna, Sweden; 35 Center for Psychiatric Research, Stockholm City Council, Stockholm, Sweden; 36 Department of Pediatrics, University of Montreal, Montreal, Canada; 37 Department of Clinical and Developmental Neuropsychology, University of Groningen, Groningen, Netherlands; 38 Department of Psychiatry, School of Medicine, Yale University, New Haven, CT United States; 39 Department of Psychiatry, Icahn School of Medicine at Mount Sinai, New York, NY United States; 40 Department of Radiation Oncology, University of Groningen, University Medical Center Groningen, Groningen, The Netherlands; 41 Department of Epidemiology, University of Groningen, University Medical Center Groningen, Groningen, The Netherlands; 42 Department of Neuroscience, Research Group Psychiatry, Center for Clinical Psychiatry, Leuven, Belgium

**Keywords:** Personalized medicine, Clinical genetics, Pharmacogenomics

## Abstract

Clozapine is the most effective antipsychotic for patients with treatment-resistant schizophrenia. However, response is highly variable and possible genetic underpinnings of this variability remain unknown. Here, we performed polygenic risk score (PRS) analyses to estimate the amount of variance in symptom severity among clozapine-treated patients explained by PRSs (R2) and examined the association between symptom severity and genotype-predicted CYP1A2, CYP2D6, and CYP2C19 enzyme activity. Genome-wide association (GWA) analyses were performed to explore loci associated with symptom severity. A multicenter cohort of 804 patients (after quality control *N* = 684) with schizophrenia spectrum disorder treated with clozapine were cross-sectionally assessed using the Positive and Negative Syndrome Scale and/or the Clinical Global Impression-Severity (CGI-S) scale. GWA and PRS regression analyses were conducted. Genotype-predicted CYP1A2, CYP2D6, and CYP2C19 enzyme activities were calculated. Schizophrenia-PRS was most significantly and positively associated with low symptom severity (*p* = 1.03 × 10^−3^; R2 = 1.85). Cross-disorder-PRS was also positively associated with lower CGI-S score (*p* = 0.01; R2 = 0.81). Compared to the lowest tertile, patients in the highest schizophrenia-PRS tertile had 1.94 times (*p* = 6.84×10^−4^) increased probability of low symptom severity. Higher genotype-predicted *CYP2C19* enzyme activity was independently associated with lower symptom severity (*p* = 8.44×10^−3^). While no locus surpassed the genome-wide significance threshold, rs1923778 within *NFIB* showed a suggestive association (*p* = 3.78×10^−7^) with symptom severity. We show that high schizophrenia-PRS and genotype-predicted CYP2C19 enzyme activity are independently associated with lower symptom severity among individuals treated with clozapine. Our findings open avenues for future pharmacogenomic projects investigating the potential of PRS and genotype-predicted CYP*-*activity in schizophrenia.

## Introduction

About one-third of patients with schizophrenia is considered to have treatment-resistant schizophrenia (TRS) [1]. TRS has been defined as the persistence of symptoms despite at least two trials of antipsychotic medications of adequate dose and duration with documented adherence [2–4]. For patients with TRS, clozapine is the most effective antipsychotic drug [5, 6]. However, 40% of TRS-patients achieve no sufficient response to clozapine, suggesting that up to 20% of schizophrenia patients are ultra-resistant (defined as failure to respond to adequate trials of two antipsychotics and clozapine) [7]. Despite its efficacy, the mean delay in clozapine prescription reaches up to 9 years [8–10], which in turn is associated with poor treatment outcomes and less functional recovery [11, 12]. Elucidating determinants of symptom severity while on clozapine may contribute to early identification of those more likely to be responsive to clozapine, enabling patients to start clozapine earlier in their disease course, resulting in a better quality of life, increased life expectancy, and lower economic burden [13, 14]. This is all the more important given the evidence that clozapine as first- or second-line therapy is more effective than other antipsychotics [11, 15, 16]. In addition, patients who are less likely to be responsive to clozapine may delay clozapine treatment, avoiding unnecessary potential side effects and blood monitoring.

A promising strategy for the identification of genetic variation associated with symptom severity among clozapine users, is the genome-wide association study (GWAS) that also allows to generate data for polygenic risk scoring (PRS). Several GWASs have identified candidate loci for clozapine blood concentrations and severe adverse drug reactions associated with clozapine, but limited focus has been given to symptomatic outcomes in patients using clozapine [17–21]. To our knowledge, there has only been one study that used a genome-wide approach to examine clozapine treatment outcome [22]. In that study of 123 clozapine-treated individuals, no statistically significant differences in schizophrenia-PRS between responders and nonresponders were detected (*N* = 123, *p* = 0.06) [22]. PRS analyses performed to uncover differences between responders and nonresponders of other antipsychotics have yielded inconsistent results [23–26].

Another promising approach that complements GWAS and PRS, is the examination of known haplotype variation in genes associated with clozapine metabolism. CYP1A2 is considered the primary metabolism pathway for clozapine with secondary minor contributions from CYP3A4/5, CYP2D6, and CYP2C19 that collectively produce two metabolites, norclozapine (active) and clozapine n-oxide (inactive) [27]. Although norclozapine possesses the hallmark characteristics of an atypical antipsychotic [28], it is thought to have little antipsychotic activity and may be responsible for side effects [29]. The role CYP enzymes play in clozapine metabolism can be exemplified by the comparison of clozapine blood concentrations of smokers and nonsmokers. The polycyclic aromatic hydrocarbons produced by smoking are established inducers of CYP1A2 enzymatic activity and smokers have consistently been shown to have lower clozapine and higher norclozapine blood concentrations compared to nonsmokers [30, 31]. Thus, the relationship between CYP enzyme activity and clozapine metabolism is notable, can differ between persons, and as a result, clozapine blood concentrations among individuals prescribed the same dose may vary. Because of this variation, therapeutic drug monitoring of clozapine blood concentrations is routinely applied. Previous studies have suggested that clozapine blood concentrations ≥350 ng/mL are associated with superior symptomatic outcome [32]. However, determining the required dose for an individual to achieve this target blood concentration and symptomatic relief can be challenging. As such, several studies have used haplotype variation in *CYP1A2, CYP2C19*, and *CYP2D6* as markers of an individual’s capacity to metabolize clozapine and their probability of receiving symptomatic relief [33–36]. Although results of these studies have been inconsistent, recent findings suggest these inconsistencies could be a result of phenoconversion, a phenomenon in which an individual’s genotype-predicted drug metabolism does not reflect their observed metabolism, due to the presence of nongenetic factors such as concomitant medications (e.g., (es)citalopram) and smoking behavior [37]. Thus, we hypothesized that genotype-predicted drug metabolism corrected for phenoconversion would be associated with clozapine blood concentrations and symptom severity.

Given the lack of established knowledge about genetic mechanisms underlying clozapine treatment outcome, this is the first study in patients with severe schizophrenia that dissects associations between symptom severity and genome-wide data. An international team with a range of different backgrounds joined forces several years ago, resulting in a unique and the largest dataset of clozapine users with genome-wide and symptom severity data available. The aims of the study were to analyze the amount of variance in symptom severity among clozapine-treated patients explained by PRSs, to examine the association between symptom severity and genotype-predicted *CYP1A2*, *CYP2D6*, and *CYP2C19* enzyme activity, and explore loci associated with symptom severity using GWA analyses.

## Participants and methods

### Participants

Participants (*N* = 804) came from five independent cohorts: 470 participants were recruited by the Clozapine International (CLOZIN) [38–41] consortium in the Netherlands, Germany, Austria, and Finland; 174 participants by the Genetic Risk and Outcome of Psychosis (GROUP) consortium in the Netherlands; 80 participants by the Cooperative Research Centre (CRC) in Australia; 50 participants by Hacettepe University in Turkey; and 30 participants by Mental Health Services Rivierduinen in the Netherlands. All studies were approved by their respective local Institutional Review Boards and all participants provided written informed consent prior to participation. The studies were compliant with the Declaration of Helsinki (2013) [42]. Participants were included if they: (1) were aged 18 years or older, (2) had a primary diagnosis of schizophrenia spectrum disorder (SSD), and (3) were using clozapine (no minimum duration of treatment). The eligibility criteria were not strict to represent ‘real world’ patients, as this is valuable for clinical value and applicability. Clozapine blood levels were measured in local accredited laboratories, ~12 h after the last clozapine dose intake. More information about each cohort is provided in the Supplementary Methods.

### Phenotyping

Symptom severity was assessed by treating physicians or trained study raters using the Clinical Global Impression-Severity (CGI-S) scale and/or the Positive and Negative Syndrome Scale (PANSS) [43, 44]. CGI-S scores were available for participants from CLOZIN, Hacettepe University, and Mental Health Services Rivierduinen, and PANSS scores for participants from GROUP and CRC Australia. Previous studies have shown a correlation between PANSS scores, Brief Psychiatric Rating Scale (BPRS) scores, and CGI-scores and the authors of these studies provided us with a table to convert PANSS scores to CGI-S scores (Supplementary Table 1) [45, 46]. Our main outcome was symptom severity defined as a quantitative measure (CGI-S score) and our secondary outcome was symptom severity defined as a binary measure (low vs. high symptom severity). Low symptom severity corresponded to a (converted) CGI-S score of 1 to 3 (‘normal’ to ‘mildly ill’) and high symptom severity corresponded to a (converted) CGI-S score of 4 to 7 (‘moderately ill’ to ‘among the most extremely ill patients’). See Supplementary Methods for more detailed information.

### Genotyping and quality control

Genotyping and quality control followed standard procedures and are described in Supplementary Methods.

### Statistical analysis

#### Genome-wide association analyses

Statistical analyses were conducted using PLINK v1.90b3z 64-bit and R version 3.2.2 (14 Aug 2015; http://www.r-project.org/) software packages. Explorative GWAS was conducted using linear regression for quantitative outcome and logistic regression for binary outcome. The quantitative outcome analysis was thus a quantitative trait locus (QTL) analysis, conducted similarly to previously reported [47, 48]. We performed GWA analyses of both outcomes, correcting for sex, age, age-squared, and the first 10 genetic-ancestry principal components (PCs). These analyses were conducted in our entire multi-ethnic cohort to assure diversity and inclusiveness of non-North Western European people [49]. However, to evaluate the robustness of our findings, as sensitivity analyses, we repeated all analyses after removing participants deviating more than 3 standard deviations (SD) from the means of the first four PCs, based on the HapMap3 HRC r1.1 2016 (GRCh37/hg19) population (*N* = 1397; Supplementary Fig. 1). Genome-wide significance was set at *p* < 5×10^−8^ and suggestive significance at *p* < 5×10^−5^.

Post-GWAS analysis was performed for identification and annotation of independent associations within our data, using Functional Mapping and Annotation of genetic associations (FUMA) [50] and Hi-C coupled Multi-marker Analysis of GenoMic Annotation (H-MAGMA v1.08; Supplementary Methods) [51].

#### Polygenic risk score analyses

PRS is an estimate of an individual’s polygenic liability to a certain trait [52]. PRSs were calculated for the following three traits relevant for schizophrenia or clozapine metabolism, using the most recent GWAS’ summary statistics: schizophrenia [53], cross-disorder [54], and clozapine metabolism [17]. The PRSs were corrected for sex, age, and 10 PCs, and the Bonferroni-corrected significance level was *p* < 0.017 (see Supplementary Methods and Supplementary Table 2 for details).

#### Genotype-predicted enzyme activity score analysis

Multiple drug metabolizing enzymes contribute to the demethylation and oxidation of clozapine (physiologically active compound) to N-desmethylclozapine (“norclozapine”; putatively active metabolite) and clozapine n-oxide (considered to be an inactive metabolite) [27, 55, 56]. However, *CYP1A2*, and to a lesser extent *CYP2C19* and *CYP2D6*, are considered the primary clozapine metabolizing enzymes in vivo [27]. Imputed genotype data (see ‘Genotyping—*Genotyping and quality control’*) was subjected to Stargazer v1.08 [57] to call *CYP*-haplotypes (star alleles). Activity scores for *CYP2D6* were based on translation tables maintained by the Pharmacogene Variation (PharmVar) Consortium [58] and the Pharmacogenomics Knowledgebase (PharmGKB), whereas activity scores for *CYP1A2* and *CYP2C19* followed previously published scoring methods (Supplementary Methods and Supplementary Table 4) [59–61].

Prior to analysis, *CYP-*activity scores were corrected for concomitant inhibitors or inducers of each of the corresponding genes using previously employed phenoconversion methodology (Supplementary Methods and Supplementary Table 5) [37]. To determine if *CYP-*activity scores were associated with symptom severity outcomes, logistic and linear regression models were fitted, with age, sex, dose-adjusted clozapine levels (i.e., concentration-to-dose ratio, one measurement per participant), and duration of clozapine therapy included as covariates [37]. In addition, linear regression models were fitted to estimate the amount of variance in dose-adjusted clozapine levels that was explained by *CYP2C19*, *CYP1A2*, and *CYP2D6*. Levels of N-desmethylclozapine were unavailable. The Bonferroni-corrected significance level was *p* < 0.017, as we corrected for three independent regression analyses performed.

In the event of detection of significant associations between symptom severity on the one and PRS as well as CYP genotypes on the other hand, we hypothesized these were independent and therefore tested whether this was indeed the case by adding the significant PRS(s) to the model.

## Results

### Genome-wide association analysis

Six hundred and eight-four individuals and 5,506,411 SNPs passed the QC and were included in the GWAS. There were 330 participants with low and 354 with high symptom severity (see Supplementary Table 6 for demographic and clinical characteristics). Linkage disequilibrium (LD)-intercept scores and genomic inflation correction factors (λGC) pre- and postimputation were all <1.02, suggesting no inflation of the test statistics (Supplementary Fig. 2).

**Fig. 1:**
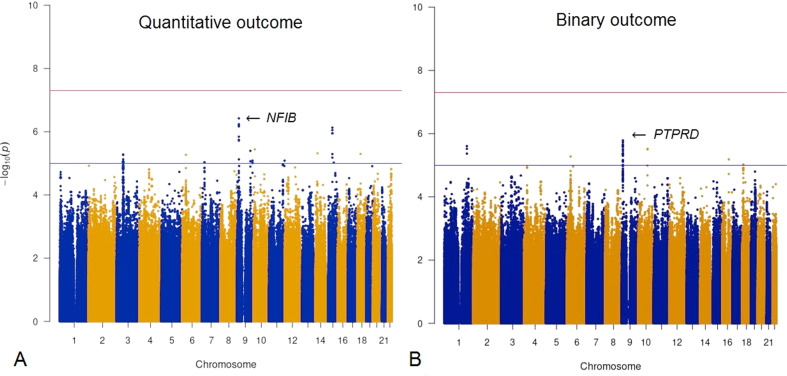
Genome-wide association analysis of symptom severity while on clozapine. **A, B** Manhattan plots depicting the genome-wide association results of symptom severity while on clozapine for quantitative and binary outcome. The *X*-axis shows the chromosomal positions. The *Y*-axis shows –log10 (*p* values). The red line illustrates the genome-wide significance level of *p* = 5 × 10^−8^, and the blue line illustrates the suggestive level of significance of *p* = 5 × 10^−5^. The arrows indicate the top loci and the closest genes.

No genome-wide significant hits were identified. The most significantly associated locus was detected between quantitative outcome and intronic rs1923778 on chromosome 9 (*p* = 3.78×10^−7^; Fig. 1A, Supplementary Fig. 3A & Supplementary Table 7), within *nuclear factor 1 B-type (NFIB)*. We did not detect dose-adjusted clozapine level differences between genotype groups at this locus, rendering it unlikely that the association we found is merely a proxy for association with dose-adjusted clozapine levels (Supplementary Fig. 4 & Supplementary Table 8). The most significant locus for binary outcome was intronic rs4742565 on chromosome 9 (*p* = 1.64 x 10^−6^; Fig. 1B, Supplementary Fig. 3B and Supplementary Table 7), within *protein tyrosine phosphatase receptor type D (PTPRD)*. In sensitivity analyses, results remained similar (Supplementary Results). LD between the top quantitative locus and the top binary locus was R^2^ = 0.0010, D’ = 0.092 and the loci were 5 Mb apart.

Post-GWAS analyses using FUMA indicated significantly enriched differentially expressed gene sets for the hypothalamus and hippocampus for quantitative outcome (Supplementary Results, Supplementary Fig. 5A) and expression in the brain of the prioritized genes for binary outcome (Supplementary Fig. 5B). We did not find any significantly associated target genes using H-MAGMA (Supplementary Fig. 6A, B).

### Polygenic risk score analysis

**Fig. 2:**
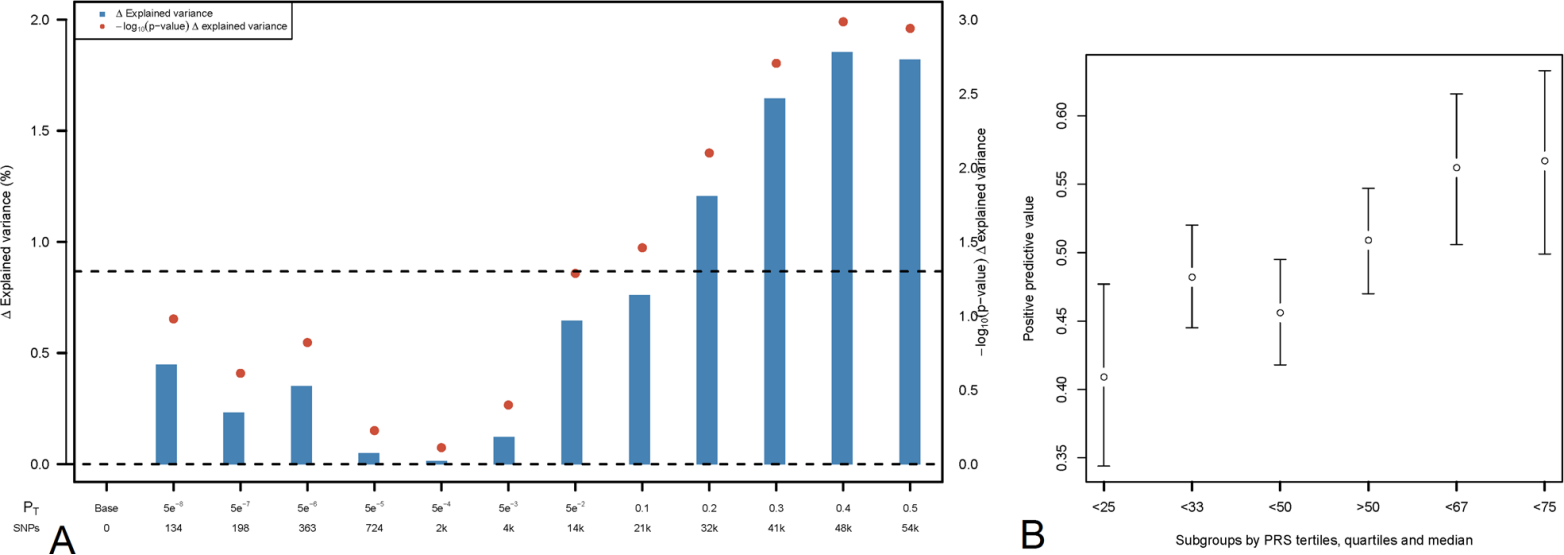
Association between symptom severity while on clozapine and polygenic risk scores for schizophrenia. **A, B**. Bar plot illustrating the explained variance for the association of schizophrenia-PRS with binary outcome at several p_t_, adjusted for sex, age, and 10 PCs. *p*_t_ are displayed on the X-axis, where the number of included SNPs increases with more lenient *p*_t_. Δ Explained variance represents the Nagelkerke R^2^ (shown as %). The red dots represent the significance of the association results (-Log 10 *p* value). The dashed line represents a nominal significance-level of *p* < 0.05 (**A**). Individual risk prediction: higher schizophrenia-PRS was associated with higher positive predictive value for low symptom severity. Whiskers represent confidence intervals (±1.96 × standard error) around the central positive predictive value estimate (**B**).

**Fig. 3:**
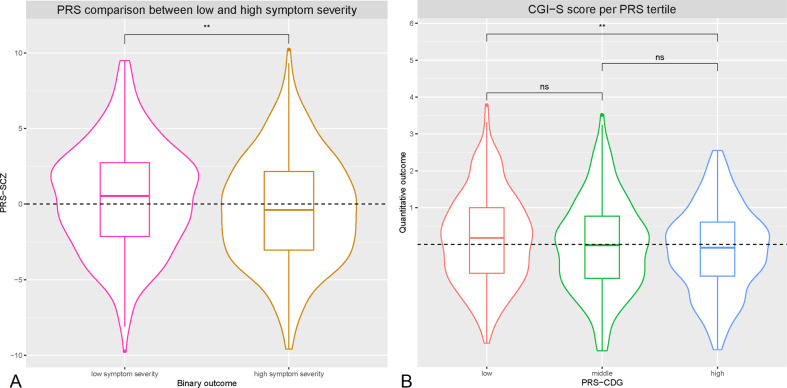
Violin plots displaying the distribution in symptom severity while on clozapine by polygenic risk score subgroups. **A, B** Violin plots of schizophrenia-PRS (PRS-SCZ) comparison for binary outcome, and cross-disorder-PRS (PRS-CDG) tertile comparison for quantitative outcome. For both analyses the best fitting *p* was used. The dashed line illustrates the mean PRS-SCZ (**A**) and the mean residual CGI-S score (**B**) in all participants. Differences were determined by linear regression of quantitative outcome on PRS tertile and using a *T*-test for binary outcome, corrected for sex, age, and 10 PCs (Supplementary Methods). **p < 5.0 × 10^−3^. ns nonsignificant.

The same 684 individuals were included in PRS-analyses. Schizophrenia-PRS was significantly associated with binary outcome (*p* = 1.03x10^−3^, *R*^2^ = 1.85, *p*_t_ = 0.4, Figs. 2A and 3A). Patients in the highest schizophrenia-PRS tertile had 1.94 times (*p* = 6.84x10^−4^, 95% confidence interval (CI) = 1.33–2.81) increased chances of low symptom severity while on clozapine compared to patients in the lowest schizophrenia-PRS tertile (Fig. 2B, Supplementary Table 9 and Supplementary Table 10). Similarly, patients in the highest schizophrenia-PRS decile had 2.26 times (*p* = 3.96x10^−3^, 95% CI = 1.30–3.91) increased chances of low symptom severity while on clozapine compared to patients in the lowest schizophrenia-PRS decile (Fig. 2B, Supplementary Table 9 and Supplementary Table 10). Cross-disorder-PRS was significantly associated with quantitative outcome (*p* = 0.01, *R*^2^ = 0.81, p_t_ = 0.3, Fig. 3B). Other PRS association results were not significant (Supplementary Results, Supplementary Fig. 7A–F). In sensitivity analyses results remained similar (Supplementary Results).

### Genotype-predicted enzyme activity score analyses

**Fig. 4:**
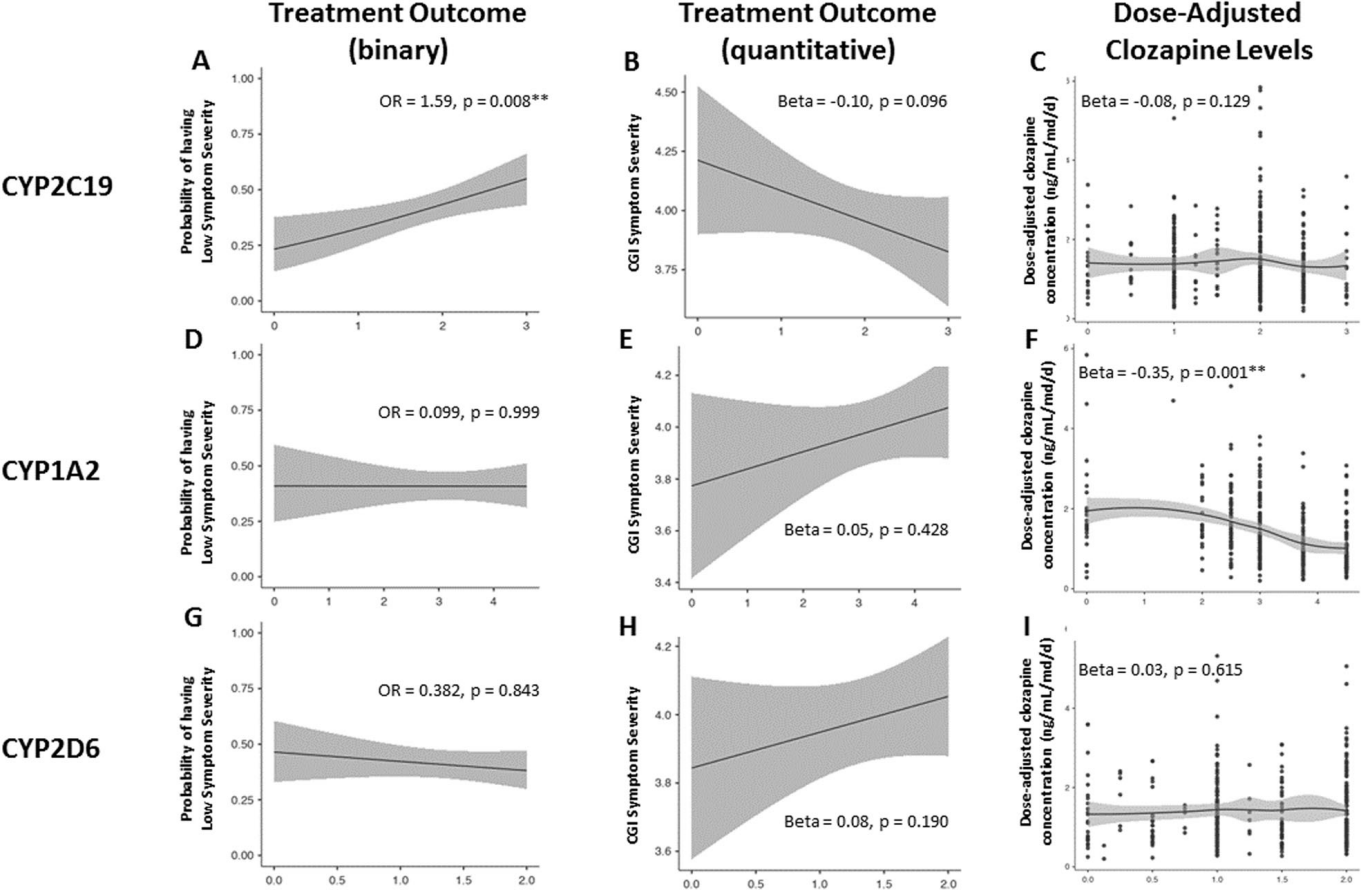
Association between *CYP*-activity scores, symptom severity while on clozapine and dose-adjusted clozapine levels. **A**–**I**. Association of corrected CYP2C19*,* CYP1A2, and CYP2D6 genotype-predicted activity scores with symptom severity while on clozapine and dose-adjusted clozapine levels.

Higher *CYP2C19* activity score was significantly associated with greater probability of low symptom severity (i.e., symptom improvement) (odds ratio (OR) = 1.59, 95% CI = 1.13–2.24, *p* = 8.44x10^−3^, *N* = 291, Supplementary Table 11, Fig. 4A) and quantitatively lower symptom severity scores (beta = −0.10, *p* = 0.10; Supplementary Table 12 and Fig. 4B) but was not associated with dose-adjusted clozapine levels (Supplementary Table 13, Fig. 4C). The association between *CYP2C19* activity score and the probability of low symptom severity did not change when including schizophrenia-PRS (OR = 1.59, 95% CI = 1.13–2.24, *p* = 8.51x10^−3^, Supplementary Table 14), schizophrenia-PRS and the first 10 PCs (OR = 1.63, 95% CI = 1.97–2.43, *p* = 0.02, Supplementary Table 15), or the top two GWAS hits (*NFIB* rs1923778, *PTPRD* rs4742565) (OR = 1.60, 95% CI = 1.06–2.42, *p* = 0.02, Supplementary Table 16).

*CYP1A2* activity score was not associated with our binary (Fig. 4D) or quantitative (Fig. 4E) outcomes but was inversely correlated with dose-adjusted clozapine levels (beta = −0.35, *p* = 2.71x10^−10^; Supplementary Table 13, Fig. 4F). *CYP2D6* activity score was not associated with either symptom severity or dose-adjusted clozapine levels (Fig. 4G–I). Neither *NFIB* rs1923778 (beta = −0.08, *p* = 0.47) nor *PTPRD* rs4742565 (beta = 0.07, *p* = 0.26) were associated with dose-adjusted clozapine levels. No interaction between *CYP2C19* and schizophrenia-PRS was found for binary outcome (beta = 0.04, *p* = 0.17). It is important to note that the C/D ratios were significantly higher in the Hacettepe cohort, compared to the other cohorts (Supplementary Table 8). In sensitivity analyses results remained similar, indicating that ancestry does not influence the results and conclusions (Supplementary Results). Furthermore, there was no difference in the dose-adjusted clozapine concentrations between people with low symptom severity (mean = 1.37, SD = 0.86) and high symptom severity (mean = 1.42, SD = 0.91; *t* = 0.57, *p* = 0.57; Supplementary Results, Supplementary Table 17&18, Supplementary Fig. 13) or in absolute clozapine concentrations between people with low symptom severity (mean = 426.35, SD = 261.12) and high symptom severity (mean = 421.67, SD = 239.30; *t* = −0.17, p = 0.88; Supplementary Fig. 13).

## Discussion

To our knowledge, this is the first study using a comprehensive, genome-wide approach to examine the genomic underpinnings of symptom severity among individuals with SSD treated with clozapine (*N* = 804 before and *N* = 684 after QC). Using a novel approach of integrating genome-wide, PRS, and *CYP* analyses, we demonstrate that higher schizophrenia-PRS and higher genotype-predicted CYP2C19 enzyme activity are independently associated with lower symptom severity while on clozapine.

Although no significant genome-wide hit was discovered, the loci on *NFIB* (rs1923778, *p* = 3.78x10^−7^) and *PTPRD* (rs4742565, *p* = 1.64x10^−6^) are of interest given previous literature. *NFIB* is a protein-coding gene associated with embryonic development, tumor growth, and brain development [62]. A recent GWAS of clozapine levels found a different locus on *NFIB* (rs28379954; Supplementary Table 19) to be associated with clozapine levels (*p* = 1.68×10^−8^) and risk of subtherapeutic serum concentrations [17]. Moreover, *NFIB* expression is correlated with clinical improvement in depressed patients treated with citalopram, suggesting it could be of interest for pharmacogenomic studies in psychiatry more broadly than merely for antidepressants [63]. *PTPRD* is a protein-coding gene that is highly expressed in the brain and that plays a role in synaptic adhesion and organization and has been shown to regulate neurogenesis in mice [64]. Several studies have shown that *PTPRD* has both an oligogenic and a polygenic contribution of common and rare variations in neurological, behavioral and neurodevelopmental disorders and was found to be associated with schizophrenia spectrum disorder, obsessive-compulsive disorder, and substance use disorders [64–69]. No previous association between *PTPRD* and clozapine has been reported, but two recent GWASs found *PTPRD* to be associated with antipsychotic-induced weight gain (*p* = 9.26 × 10-9) [70] and suggestively associated with response to the atypical antipsychotic lurasidone in European and African participants (*p* = 6.03 × 10-5 and *p* = 4.29 x 10−5, respectively) [23].

Our PRS analyses revealed that higher schizophrenia-PRS was associated with lower symptom severity as a binary trait and that higher cross-disorder-PRS was associated with lower symptom severity as a quantitative trait. To our knowledge, no previous study has examined the association between cross-disorder-PRS and antipsychotic treatment outcome. However, several studies have evaluated the association between schizophrenia-PRS and antipsychotic treatment outcome [22–26], although only one of these studies examined clozapine response [22]. In line with our results, that study reported higher schizophrenia-PRS in clozapine responders compared to clozapine nonresponders, although this was not significant (*p* = 0.06) [22]. Differences in phenotyping (here we used PANSS and CGI-S scales; in the previous study a nonvalidated, 4-level ordinal scale was used) and power (*N* = 684 vs. *N* = 123) possibly explain differences in statistical significance (*p* = 1.03 × 10^−3^ vs. *p* = 0.06). The same direction of association was found in a GWAS of lurasidone response (*N* = 429) [23] and a risperidone response GWAS [24]. However, a study with first-episode psychosis patients using several antipsychotics (but not clozapine) found that higher schizophrenia-PRS was associated with lower response rates (i.e. higher symptom severity) [71]. Several studies indicate that schizophrenia-PRS increases when comparing first-episode psychosis to schizophrenia, and schizophrenia to TRS [22, 71–73], although there is also conflicting evidence for the latter [74, 75]. Bearing those former observations in mind, higher schizophrenia-PRS may characterize a subset of TRS-patients more likely to respond well to clozapine. Speculatively, people with low schizophrenia-PRS may thus have a better prognosis with shorter duration of illness, whereas in advanced stages of illness those with high schizophrenia-PRS may respond better to clozapine [73]. Alternatively, it is also plausible that people with higher schizophrenia-PRS are genetically more close to schizophrenia and therefore more responsive to clozapine, while people who are more genetically distant and/or where other environmental factors exert an influence are less responsive to clozapine.

Furthermore, we found a positive association between genotype-predicted CYP2C19 enzyme activity and symptom severity. This finding aligns with a previous study of 137 clozapine-treated patients that reported *CYP2C19* ultrarapid metabolizers (*17/*17) were five times more likely to show clinical improvements [76], although smaller studies have shown no association [37, 77] or an inverse association [78] between CYP2C19*17 carriers and clozapine-related symptomatic outcomes. Although *CYP2C19* is involved in the demethylation of clozapine to N-desmethylclozapine, a pharmacologically active metabolite that binds to an array of receptors including dopamine D2 and D3 receptors, muscarinic receptors and serotonergic receptors [27], current evidence suggests *CYP2C19* genetic variation has limited effect on clozapine metabolism [37, 79, 80]. As such, the association between CYP2C19 enzymatic activity and improvement in clinical outcome is unlikely to be explained by differential clozapine blood concentrations. In fact, we did not detect an association between CYP2C19 enzyme activity and clozapine concentrations. Alternatively, our findings may reflect CYP2C19’s role in metabolizing endogenous compounds in the brain [81]. Human studies have shown increased CYP2C19 genotype-predicted activity was associated with smaller hippocampi volumes and greater suicidality [82], although attempts to replicate these associations have been unsuccessful [83]. A weakness of our study is the lack of a replication cohort and consequently our findings await replication in larger datasets with diverse ancestries to more firmly guard against the risk of type-I error that is inherent in research projects (such as ours) where several statistical tests are performed. Therefore, future investigations of the relationships between genotype-predicted CYP2C19 enzyme activity and symptomatic outcomes are warranted. In such future studies, norclozapine should also be taken into account to allow a separate genetic study on such levels and ratios with clozapine. Additionally, it is interesting that in the current study no association between *CYP1A2* and symptom severity is found, as this was suggested from a previous study [37]. Perhaps this difference in findings is explained by the use of different measurements of symptom severity, as the previous study used PANSS [37], while the current study used CGI-S which is less specifically about psychotic symptoms. It is good to note, there is still little understanding of the relationship between *CYP1A2* and symptom severity, and this should be investigated in more detail.

Strengths of our study include our unique sample of over 800 multiethnic patients with SSD using clozapine, and the use of a very recent GWAS-platform, and genotype-predicted activity of relevant metabolizing enzymes. Furthermore, although no longitudinal data on clozapine response rates was available for our participants, the distribution of low and high symptom severity while on clozapine in our cohort (*N* = 330 vs. *N* = 354, respectively) aligns with reported response rates to clozapine [7]. Nonetheless, several limitations should be considered when interpreting our results. First, the main limitation is that our phenotype, symptom severity, was assessed cross-sectionally and therefore no distinction can be made between clozapine response and individual variability in disease severity. In general, patients starting clozapine have a fairly severe disease course with high symptom severity prior to initiation of treatment. However, there is a high degree of heterogeneity in symptom severity in this population and data on symptom severity prior to initiation of clozapine was lacking, so the results of the GWAS should be interpreted in light of this limitation. Second, participants were cross-sectionally ascertained using two symptom scales, which made the use of a conversion table necessary. The lack of inflation of our GWAS test statistic as well as the consistency between the results of the main and sensitivity analyses’ nonetheless support the robustness of the results. Third, our study population is derived from multiple cohorts and not all covariate data was complete for all cohorts. For future GWASs, it would be highly interesting to perform subgroup analyses on patients stopping clozapine due to poor clinical effect, as this could provide highly valuable genetic information on ultra-TRS, and to examine the effects of possible gender differences, previous antipsychotic medication, and concomitant therapies. We are also currently working on a genome-wide meta-analysis of clozapine blood levels to further elucidate possible genetic underlying mechanisms of the interindividual variability in clozapine concentrations. In the meantime, the recruitment of clozapine users for the CLOZIN study is still ongoing, so that we can perform a more powerful GWAS in the near future. This larger sample size is also needed to be able to detect associations between single SNPs within CYP enzymes and clozapine concentrations, as we lacked statistical power to do so. Fourth, we estimate that about 3% of our cohort were incorrectly classified as *CYP2D6* normal metabolizers due to the absence of copy number variation data required to detect *CYP2D6* ultrarapid metabolizers (Supplementary Methods). However, to our knowledge, there is no evidence suggesting this misclassification error would have a meaningful impact on our study findings. Fifth, as the GROUP cohort is an older cohort, there might be sample overlap between the current and the PGC-schizophrenia study population and consequently the PGC-cross-disorder study population. We therefore repeated our analyses excluding the samples from GROUP: all PRS association patterns remained similar with the same p_t_, albeit less significant, as expected due to loss of power (Supplementary Fig. 9A, B). Furthermore, there were no clozapine concentrations available from GROUP. Therefore, this cohort was not represented in the *CYP* analyses. Another limitation is that we could not definitely determine for all cohorts whether clozapine concentrations represented steady state and trough levels nor could we verify adherence. Nonetheless, as our observed clozapine C/D ratios follow the expected pattern of steady-state and trough levels (Supplementary Fig. 14A–D), it is likely that these levels are reliable approximations. Inflammation (e.g., C-reactive protein) and caffeine consumption data, which can inhibit CYP1A2 activity, were not available and could therefore be over-estimated for some participants. Finally, caution in the interpretation of our GWAS results is warranted given the lack of a replication cohort.

In conclusion, we demonstrate for the first time that higher schizophrenia-PRS and *CYP2C19* predicted activity are independently associated with low symptom severity among clozapine-treated schizophrenia patients. For future clinical translation, if these findings are replicated, schizophrenia-PRS and genotype-predicted CYP2C19 activity may be used in conjunction with nongenetic factors to help predict clozapine response, ultimately allowing early identification of individuals more likely responding to clozapine. Such future studies should also incorporate deeper phenotyping information collected (e.g., clinical symptomatology in further detail, steady-state clozapine levels, adherence) in a longitudinal design. Timely prescribing may improve patients’ prognosis, given clozapine’s superiority over other antipsychotics in early disease stages [15].

## Supplementary information

Supplemental Material

## References

[1] Lally J, MacCabe JH. Antipsychotic medication in schizophrenia: A review. Br Med Bull. 2015;114:169–79. 10.1093/bmb/ldv017 25957394

[2] Howes OD, McCutcheon R, Agid O, de Bartolomeis A, van Beveren NJM, Birnbaum ML (2017). Treatment-Resistant Schizophrenia: Treatment Response and Resistance in Psychosis (TRRIP) Working Group Consensus Guidelines on Diagnosis and Terminology. Am J Psychiatry.

[3] Kane JM, Leucht S, Carpenter D, Docherty JP (2003). Expert consensus guideline series. Optimizing pharmacologic treatment of psychotic disorders. Introduction: methods, commentary, and summary. J Clin Psychiatry..

[4] Kreyenbuhl J, Buchanan RW, Dickerson FB, Dixon LB (2010). The schizophrenia patient outcomes research team (PORT): Updated treatment recommendations 2009. Schizophr Bull.

[5] Luykx JJ, Stam N, Tanskanen A, Tiihonen J, Taipale H. In the aftermath of clozapine discontinuation: comparative effectiveness and safety of antipsychotics in patients with schizophrenia who discontinue clozapine. Br J Psychiatry. 2020;217:498–505. 10.1192/bjp.2019.267 PMC7511905 31910911

[6] Miyamoto S, Miyake N, Jarskog LF, Fleischhacker WW, Lieberman JA. Pharmacological treatment of schizophrenia: A critical review of the pharmacology and clinical effects of current and future therapeutic agents. Mol Psychiatry. 2012;17:1206–27. 10.1038/mp.2012.47 22584864

[7] Siskind D, Siskind V, Kisely S (2017). Clozapine Response Rates among People with Treatment-Resistant Schizophrenia: Data from a Systematic Review and Meta-Analysis. Can J Psychiatry.

[8] Taylor DM, Young C, Paton C (2003). Prior antipsychotic prescribing in patients currently receiving clozapine: A case note review. J Clin Psychiatry.

[9] Doyle R, Behan C, OʼKeeffe D, Masterson S, Kinsella A, Kelly A, et al. Clozapine Use in a Cohort of First-Episode Psychosis. J Clin Psychopharmacol. 2017;37:512–7. 10.1097/JCP.0000000000000734 28650930

[10] Howes OD, Vergunst F, Gee S, McGuire P, Kapur S, Taylor D (2012). Adherence to treatment guidelines in clinical practice: Study of antipsychotic treatment prior to clozapine initiation. Br J Psychiatry.

[11] Shah P, Iwata Y, Plitman E, Brown EE, Caravaggio F, Kim J, et al. The impact of delay in clozapine initiation on treatment outcomes in patients with treatment-resistant schizophrenia: A systematic review. Psychiatry Res. 2018;268:114–22. 10.1016/j.psychres.2018.06.070 30015109

[12] John AP, Ko EKF, Dominic A (2018). Delayed Initiation of Clozapine Continues to Be a Substantial Clinical Concern. Can J Psychiatry.

[13] Jin H, Tappenden P, MacCabe JH, Robinson S, McCrone P, Byford S Cost and health impacts of adherence to the National Institute for Health and Care Excellence schizophrenia guideline recommendations. Br J Psychiatry. 2020:1–6. 10.1192/bjp.2020.241 33308329

[14] Vermeulen JM, van Rooijen G, van de Kerkhof MPJ, Sutterland AL, Correll CU (2019). de Haan L. Clozapine and Long-Term Mortality Risk in Patients With Schizophrenia: A Systematic Review and Meta-analysis of Studies Lasting 1.1-12.5 Years. Schizophr Bull.

[15] Okhuijsen-Pfeifer C, Huijsman EAH, Hasan A, Sommer IEC, Leucht S, Kahn RS (2018). Clozapine as a first- or second-line treatment in schizophrenia: a systematic review and meta-analysis. Acta Psychiatr Scand.

[16] Siskind D, McCartney L, Goldschlager R, Kisely S (2016). Clozapine v. first- and second-generation antipsychotics in treatment-refractory schizophrenia: systematic review and meta-analysis. Br J Psychiatry.

[17] Smith RL, O’Connell K, Athanasiu L, Djurovic S, Kringen MK, Andreassen OA (2020). Identification of a novel polymorphism associated with reduced clozapine concentration in schizophrenia patients-a genome-wide association study adjusting for smoking habits. Transl Psychiatry.

[18] Pardiñas AF, Nalmpanti M, Pocklington AJ, Legge SE, Medway C, King A (2019). Pharmacogenomic Variants and Drug Interactions Identified Through the Genetic Analysis of Clozapine Metabolism. Am J Psychiatry.

[19] de With SAJ, Pulit SL, Staal WG, Kahn RS, Ophoff RA (2017). More than 25 years of genetic studies of clozapine-induced agranulocytosis. Pharmacogenomics J.

[20] Legge SE, Hamshere ML, Ripke S, Pardinas AF, Goldstein JI, Rees E (2018). Genome-wide common and rare variant analysis provides novel insights into clozapine-associated neutropenia. Mol Psychiatry.

[21] Legge SE, Pardiñas AF, Helthuis M, Jansen JA, Kollie K, Knapper S (2019). A genome-wide association study in individuals of African ancestry reveals the importance of the Duffy-null genotype in the assessment of clozapine-related neutropenia. Mol Psychiatry.

[22] Frank J, Lang M, Witt SH, Strohmaier J, Rujescu D, Cichon S, et al. Identification of increased genetic risk scores for schizophrenia in treatment-resistant patients. Mol Psychiatry. 2015. 10.1038/mp.2014.56. 10.1038/mp.2015.52 25869806

[23] Li J, Yoshikawa A, Brennan MD, Ramsey TL, Meltzer HY (2018). Genetic predictors of antipsychotic response to lurasidone identified in a genome wide association study and by schizophrenia risk genes. Schizophr Res.

[24] Santoro ML, Ota V, de Jong S, Noto C, Spindola LM, Talarico F (2018). Polygenic risk score analyses of symptoms and treatment response in an antipsychotic-naive first episode of psychosis cohort. Transl Psychiatry.

[25] Amare AT, Schubert KO, Hou L, Clark SR, Papiol S, Heilbronner U (2018). Association of Polygenic Score for Schizophrenia and HLA Antigen and Inflammation Genes With Response to Lithium in Bipolar Affective Disorder: A Genome-Wide Association Study. JAMA Psychiatry.

[26] Zhang J-P, Robinson D, Yu J, Gallego J, Fleischhacker WW, Kahn RS (2019). Schizophrenia Polygenic Risk Score as a Predictor of Antipsychotic Efficacy in First-Episode Psychosis. Am J Psychiatry.

[27] Thorn CF, Müller DJ, Altman RB, Klein TE (2018). PharmGKB summary: clozapine pathway, pharmacokinetics. Pharmacogenet Genomics.

[28] Lameh J, Burstein ES, Taylor E, Weiner DM, Vanover KE, Bonhaus DW (2007). Pharmacology of N-desmethylclozapine. Pharm Ther.

[29] Mendoza MC, Lindenmayer JP (2009). N-desmethylclozapine: is there evidence for its antipsychotic potential?. Clin Neuropharmacol.

[30] Seppälä NH, Leinonen EV, Lehtonen ML, Kivistö KT (1999). Clozapine serum concentrations are lower in smoking than in non-smoking schizophrenic patients. Pharm Toxicol.

[31] Meyer JM (2001). Individual changes in clozapine levels after smoking cessation: results and a predictive model. J Clin Psychopharmacol.

[32] Wagner E, Kane JM, Correll CU, Howes O, Siskind D, Honer WG, et al. Clozapine Combination and Augmentation Strategies in Patients With Schizophrenia -Recommendations From an International Expert Survey Among the Treatment Response and Resistance in Psychosis (TRRIP) Working Group. Schizophr Bull. 2020. 10.1093/schbul/sbaa060. 10.1093/schbul/sbaa060 PMC7846085 32421188

[33] Jaquenoud Sirot E, Knezevic B, Morena GP, Harenberg S, Oneda B, Crettol S (2009). ABCB1 and cytochrome P450 polymorphisms: clinical pharmacogenetics of clozapine. J Clin Psychopharmacol.

[34] Brennan MD (2014). Pharmacogenetics of second-generation antipsychotics. Pharmacogenomics.

[35] Lee S-T, Ryu S, Kim S-R, Kim S, Kim J-W, Lee S-Y (2012). Association study of 27 annotated genes for clozapine pharmacogenetics: validation of preexisting studies and identification of a new candidate gene, ABCB1, for treatment response. J Clin Psychopharmacol.

[36] Pouget JG, Shams TA, Tiwari AK, Müller DJ (2014). Pharmacogenetics and outcome with antipsychotic drugs. Dialogues Clin Neurosci.

[37] Lesche D, Mostafa S, Everall I, Pantelis C, Bousman CA (2020). Impact of CYP1A2, CYP2C19, and CYP2D6 genotype- and phenoconversion-predicted enzyme activity on clozapine exposure and symptom severity. Pharmacogenomics J.

[38] Wagner E, Oviedo-Salcedo T, Pelzer N, Strube W, Maurus I, Gutwinski S (2020). Effects of Smoking Status on Remission and Metabolic and Cognitive Outcomes in Schizophrenia Patients Treated with Clozapine. Pharmacopsychiatry.

[39] Okhuijsen-Pfeifer C, Ayhan Y, Lin BD, van Eijk KR, Bekema E, Kool LJGB, et al. Genetic Susceptibility to Clozapine-Induced Agranulocytosis/Neutropenia Across Ethnicities: Results From a New Cohort of Turkish and Other Caucasian Participants, and Meta-Analysis. Schizophr Bull Open. 2020. 10.1093/schizbullopen/sgaa024.

[40] Okhuijsen-Pfeifer C, Cohen D, Bogers JPAM, de Vos CMH, Huijsman EAH, Kahn RS (2019). Differences between physicians’ and nurse practitioners’ viewpoints on reasons for clozapine underprescription. Brain Behav.

[41] Huisman R, Okhuijsen-Pfeifer C, Mulder EYH, Jongkind A, Cohen D, Bogers, JPAM, [Validatie van de Nederlandstalige Glasgow Antipsychotica Bijwerkingen Schaal voor Clozapine]. Tijdschr Psychiatr. Published online 2020. 33913142

[42] World Medical Association. *Declaration of Helsinki. Ethical Principles for Medical Research Involving Human Subjects.64th WMA General Assembly, Fortaleza, Brazil* 2013;2013.

[43] Kay SR, Fiszbein A, Opler LA. The positive and negative syndrome scale (PANSS) for schizophrenia. Schizophr Bull. 1987;13:261–76. 10.1093/schbul/13.2.261 3616518

[44] National Institute of Mental Health. CGI. Clinical Global Impressions. Psychiatry (Edgmont). 2007;4:28–37. PMC2880930 20526405

[45] Leucht S, Kane JM, Etschel E, Kissling W, Hamann J, Engel RR. Linking the PANSS, BPRS, and CGI: Clinical implications. Neuropsychopharmacology. 2006;31:2318–25. 10.1038/sj.npp.1301147 16823384

[46] Leucht S, Rothe P, Davis JM, Engel RR. Equipercentile linking of the BPRS and the PANSS. Eur Neuropsychopharmacol. 2013;23:956–9. 10.1016/j.euroneuro.2012.11.004 23433639

[47] Luykx JJ, Bakker SC, Visser WF, Verhoeven-Duif N, Buizer-Voskamp JE, den Heijer JM (2015). Genome-wide association study of NMDA receptor coagonists in human cerebrospinal fluid and plasma. Mol Psychiatry.

[48] Luykx JJ, Bakker SC, Lentjes E, Neeleman M, Strengman E, Mentink L (2014). Genome-wide association study of monoamine metabolite levels in human cerebrospinal fluid. Mol Psychiatry.

[49] Nievergelt CM, Maihofer AX, Klengel T, Atkinson EG, Chen C-Y, Choi KW (2019). International meta-analysis of PTSD genome-wide association studies identifies sex- and ancestry-specific genetic risk loci. Nat Commun.

[50] Watanabe K, Taskesen E, van Bochoven A, Posthuma D (2017). Functional mapping and annotation of genetic associations with FUMA. Nat Commun.

[51] Sey NYA, Hu B, Mah W, Fainu H, McAfee JC, Rajarajan P (2020). A computational tool (H-MAGMA) for improved prediction of brain-disorder risk genes by incorporating brain chromatin interaction profiles. Nat Neurosci.

[52] Wray NR, Goddard ME, Visscher PM (2007). Prediction of individual genetic risk to disease from genome-wide association studies. Genome Res.

[53] Pardiñas AF, Holmans P, Pocklington AJ, Escott-Price V, Ripke S, Carrera N (2018). Common schizophrenia alleles are enriched in mutation-intolerant genes and in regions under strong background selection. Nat Genet.

[54] Genomic Relationships, Novel Loci, and Pleiotropic Mechanisms across Eight Psychiatric Disorders. Cell. 2019;179:1469–82.e11. 10.1016/j.cell.2019.11.020 PMC7077032 31835028

[55] Li Z, Huang M, Ichikawa J, Dai J, Meltzer HY. N-desmethylclozapine, a major metabolite of clozapine, increases cortical acetylcholine and dopamine release in vivo via stimulation of M1 muscarinic receptors. Neuropsychopharmacology. 2005;30:1986–95. 10.1038/sj.npp.1300768 15900318

[56] Sur C, Mallorga PJ, Wittmann M, Jacobson MA, Pascarella D, Williams JB, et al. N-desmethylclozapine, an allosteric agonist at muscarinic 1 receptor, potentiates N-methyl-D-aspartate receptor activity. Proc Natl Acad Sci USA. 2003;100:13674–9. 10.1073/pnas.1835612100 PMC263872 14595031

[57] Lee S-B, Wheeler MM, Thummel KE, Nickerson DA (2019). Calling Star Alleles With Stargazer in 28 Pharmacogenes With Whole Genome Sequences. Clin Pharm Ther.

[58] Gaedigk A, Ingelman-Sundberg M, Miller NA, Leeder JS, Whirl-Carrillo M, Klein TE (2018). The Pharmacogene Variation (PharmVar) Consortium: Incorporation of the Human Cytochrome P450 (CYP) Allele Nomenclature Database. Clin Pharm Ther.

[59] Mrazek DA, Biernacka JM, O’Kane DJ, Black JL, Cunningham JM, Drews MS (2011). CYP2C19 variation and citalopram response. Pharmacogenet Genomics.

[60] Saiz-Rodríguez M, Ochoa D, Belmonte C, Roman M, Vieira de Lara D, Zubiair P (2019). Polymorphisms in CYP1A2, CYP2C9 and ABCB1 affect agomelatine pharmacokinetics. J Psychopharmacol.

[61] Whirl-Carrillo M, McDonagh EM, Hebert JM, Gong L, Sangkuhl K, Thorn CG (2012). Pharmacogenomics knowledge for personalized medicine. Clin Pharm Ther.

[62] Steele-Perkins G, Plachez C, Butz KG, Yang G, Bachurski CJ, Kinsman SL (2005). The transcription factor gene Nfib is essential for both lung maturation and brain development. Mol Cell Biol.

[63] Barakat AK, Scholl C, Steffens M, Brandenburg K, Ising M, Lucae S (2020). Citalopram-induced pathways regulation and tentative treatment-outcome-predicting biomarkers in lymphoblastoid cell lines from depression patients. Transl Psychiatry.

[64] Uhl GR, Martinez MJ (2019). PTPRD: neurobiology, genetics, and initial pharmacology of a pleiotropic contributor to brain phenotypes. Ann N. Y Acad Sci.

[65] Liu X, Shimada T, Otowa T, Wu Y-Y, Kawamura Y, Tochigi M (2016). Genome-wide Association Study of Autism Spectrum Disorder in the East Asian Populations. Autism Res..

[66] Choucair N, Mignon-Ravix C, Cacciagli P, Ghoch JA, Fawaz A, Megarbane A (2015). Evidence that homozygous PTPRD gene microdeletion causes trigonocephaly, hearing loss, and intellectual disability. Mol Cytogenet.

[67] Revealing the complex genetic architecture of obsessive-compulsive disorder using meta-analysis. Mol Psychiatry. 2018;23:1181–8. 10.1038/mp.2017.154 PMC6660151 28761083

[68] Uhl GR, Martinez MJ, Paik P, Sulima A, Bi G-H, Iyer MR (2018). Cocaine reward is reduced by decreased expression of receptor-type protein tyrosine phosphatase D (PTPRD) and by a novel PTPRD antagonist. Proc Natl Acad Sci USA.

[69] Cox JW, Sherva RM, Lunetta KL, Johnson EC, Martin NG, Degenhardt L, et al. Genome-Wide Association Study of Opioid Cessation. J Clin Med. 2020;9:180. 10.3390/jcm9010180 PMC7019731 31936517

[70] Yu H, Wang L, Lv L, Ma C, Du B, Lu T (2016). Genome-Wide Association Study Suggested the PTPRD Polymorphisms Were Associated With Weight Gain Effects of Atypical Antipsychotic Medications. Schizophr Bull.

[71] Jonas KG, Lencz T, Li K, Malhotra AL, Perkman G, Fochtmann LJ (2019). Schizophrenia polygenic risk score and 20-year course of illness in psychotic disorders. Transl Psychiatry.

[72] Werner MCF, Wirgenes KV, Haram M, Bettella F, Lunding SH, Rodevand L (2020). Indicated association between polygenic risk score and treatment-resistance in a naturalistic sample of patients with schizophrenia spectrum disorders. Schizophr Res.

[73] Vassos E, Di Forti M, Coleman J, Iyegbe C, Prata D, Euesden J (2017). An Examination of Polygenic Score Risk Prediction in Individuals With First-Episode Psychosis. Biol Psychiatry.

[74] Wimberley T, Gasse C, Meier SM, Agerbo E, MacCabe JH, Horsdal HT (2017). Polygenic risk score for schizophrenia and treatment-resistant schizophrenia. Schizophr Bull.

[75] Legge SE, Dennison CA, Pardiñas AF, Rees E, Lynham AJ, Hopkins L, et al. Clinical indicators of treatment-resistant psychosis. Br J Psychiatry. 2015;216:259–66. 10.1192/bjp.2019.120 31155017

[76] Piatkov I, Caetano D, Assur Y, Lau SL, Coelho M, Jones T (2017). CYP2C19*17 protects against metabolic complications of clozapine treatment. World J Biol Psychiatry J World Fed Soc Biol Psychiatry.

[77] van de Bilt MT, Prado CM, Ojopi EPB, Sousa RT, Loch AA, Zanetti MV (2015). Cytochrome P450 genotypes are not associated with refractoriness to antipsychotic treatment. Schizophr Res.

[78] Rodrigues-Silva C, Semedo AT, Neri HF, da S, Vianello RP, Galaviz-Hernandez C (2020). The CYP2C19*2 and CYP2C19*17 Polymorphisms Influence Responses to Clozapine for the Treatment of Schizophrenia. Neuropsychiatr Dis Treat.

[79] Tóth K, Csukly G, Sirok D, Belic A, Kiss A, Hafra E (2017). Potential Role of Patients’ CYP3A-Status in Clozapine Pharmacokinetics. Int J Neuropsychopharmacol.

[80] Kirchheiner J, Nickchen K, Bauer M, Licinio J, Roots I, Brockmoller J (2004). Pharmacogenetics of antidepressants and antipsychotics: the contribution of allelic variations to the phenotype of drug response. Mol Psychiatry.

[81] Hedlund E, Gustafsson JA, Warner M (2001). Cytochrome P450 in the brain; a review. Curr Drug Metab.

[82] Ingelman-Sundberg M, Persson A, Jukic MM (2014). Polymorphic expression of CYP2C19 and CYP2D6 in the developing and adult human brain causing variability in cognition, risk for depression and suicide: the search for the endogenous substrates. Pharmacogenomics.

[83] Savadlou A, Arnatkeviciute A, Tiego J, Hawi Z, Bellgrove MA, Fornito A (2020). Impact of CYP2C19 genotype-predicted enzyme activity on hippocampal volume, anxiety, and depression. Psychiatry Res.

